# The sprained mind

**DOI:** 10.1016/j.isci.2025.111777

**Published:** 2025-02-06

## Abstract

RBSA Honorable Mention

## Main text

**Figure:**
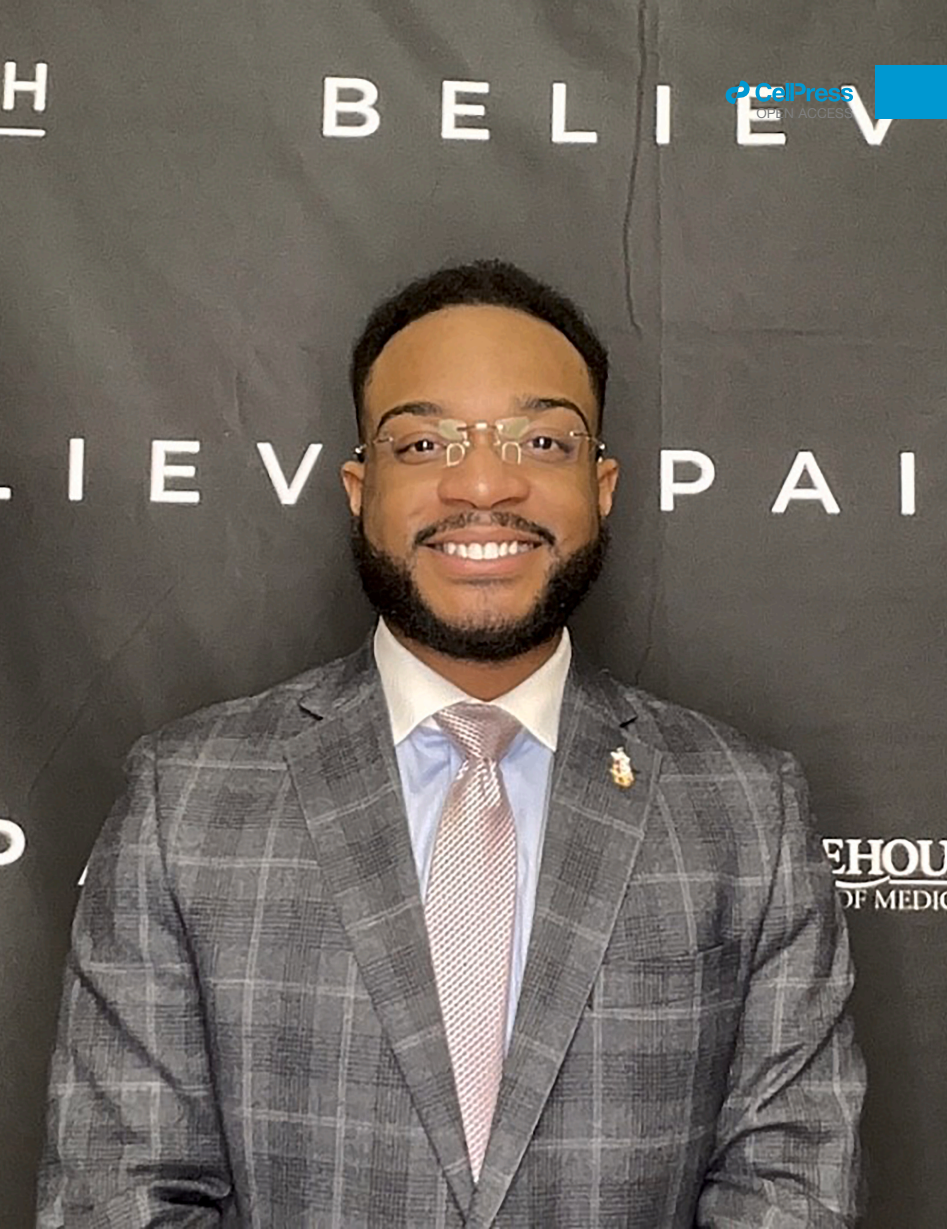
Above image: Darnell K. Adrian Williams Jr.

There’s a familiar scene we all know: someone rolls their ankle in a game of basketball. The response is clear—ice it, elevate it, and, if serious, see a doctor. No one questions the legitimacy of the injury because they can see it. I learned this contrast early when, as a young child, I watched a family member experience bipolar disorder. They faced whispers and shame in their community. This difference haunted me: how a visible injury draws compassion, while an invisible one breeds skepticism. Often in psychiatry, patients are robbed of the basic dignity of understanding their own suffering. Someone with terminal cancer can process their journey, but in psychiatry, even this awareness is often denied. This stark reality, coupled with the striking absence of objective biomarkers in psychiatric care, ignited my passion. My mission became clear: to reshape mental health care to more closely resemble the approaches we use in other fields of medicine, using science to bridge the gap between what we can and cannot see.

This reality crystallized during my time in Dr. Conor Liston’s lab at Cornell as an undergraduate student. There, observing the neural signatures of depression emerge on fMRI scans, something shifted in my understanding. These were not just abstract patterns—they were footprints of suffering, as real as any broken bone. The brain revealed its secrets in greens and reds, each pattern telling a story of struggle, resilience, and pain. These images reinforced a crucial truth: the mind is a product of a physical system—the brain and body working in concert. For the first time, I could envision a future where psychiatric diagnoses carried the same weight as an X-ray of a broken bone, where “it’s all in your head” transformed into “let me show you exactly what is happening in your brain.”

Growing up in a home where my father is African American and my mother is Guyanese, surrounded by a Nigerian community that helped raise me, I inherited rich cultural traditions—but also their complex relationship with mental health. Generations of systemic oppression created a painful paradox: survival meant suppressing emotion, leaving no space to simply feel. This enforced silence about mental health persists today, reflected in both who gets to study the mind and who receives help healing it. The statistics are stark: MD/PhDs represent only 3% of US medical graduates, but hold ∼65% of NIH-funded research grants. Within this elite group, merely 5% are Black, and even fewer are Black men. The human cost of this is devastating: suicide ranks as the third leading cause of death among African Americans aged 15–24, yet our community receives only half the mental health treatment of white Americans. These statistics are the modern manifestation of systems not built to hear our pain or heal our minds.

My drive to address these disparities runs deep. In college, as student body president and executive-director of the Ohio Student Government Association, I co-founded the “Retain the 9” initiative—a task force addressing low retention rates of minorities. The initiative established a new, permanently university-funded Office of Retention, leading to over a 10% increase in two years and helping hundreds of students remain enrolled.

During my gap year, I led a national study examining how sociodemographic factors impact MD/PhD program acceptances, collaborating with minority medical students nationwide. Our published findings illuminated systemic barriers in medical education and demonstrated how socioeconomic status affects access to medicine and science. This work strengthened my resolve to use scientific inquiry to challenge inequities and advocate for the underserved.

Now, training in the Bronx—one of America’s most diverse counties—I experience daily how mental health challenges intertwine with socioeconomic realities. My current research focuses on sub-characterizing autism spectrum disorder through neuroimaging and computational methods—searching for biological markers to make the invisible visible. Beyond the laboratory, I have created a summer science curriculum for underprivileged high schoolers and mentored over 10 minority students in developing research addressing their communities’ needs, from COVID-19’s psychiatric impact to immigration policies’ effects on maternal mental-health. For me, each mentee represents another crack in medicine’s glass ceiling.

As a future physician-scientist, my journey forward is clear—I aim to not only treat patients but to also develop culturally informed neuroimaging biomarkers for psychiatric conditions in my lab, particularly focusing on underserved communities. This work will combine advanced technology with cultural understanding to train the next generation of scientists. In addition, I aim to create pipelines for underrepresented students to enter neuroscience research and medicine.

Some might call these goals idealistic. I call them necessary. Just as we would not ignore a sprained ankle, we cannot continue to overlook “sprained minds.” I want to help build a future where every injury—seen or unseen—receives the care, compassion, and dignity it deserves, and where those studying and treating these conditions reflect the diverse communities they serve.

